# Transgenic Mice Expressing Yeast CUP1 Exhibit Increased Copper Utilization from Feeds

**DOI:** 10.1371/journal.pone.0107810

**Published:** 2014-09-29

**Authors:** Xiaoxian Xie, Yufang Ma, Zhenliang Chen, Rongrong Liao, Xiangzhe Zhang, Qishan Wang, Yuchun Pan

**Affiliations:** School of Agriculture and Biology, Department of Animal Sciences, Shanghai Jiao Tong University, Shanghai, PR China, Shanghai Key Laboratory of Veterinary Biotechnology, Shanghai, PR China; Cincinnati Children's Hospital Medical Center, United States of America

## Abstract

Copper is required for structural and catalytic properties of a variety of enzymes participating in many vital biological processes for growth and development. Feeds provide most of the copper as an essential micronutrient consumed by animals, but inorganic copper could not be utilized effectively. In the present study, we aimed to develop transgenic mouse models to test if copper utilization will be increased by providing the animals with an exogenous gene for generation of copper chelatin in saliva. Considering that the *S*. *cerevisiae CUP1* gene encodes a Cys-rich protein that can bind copper as specifically as copper chelatin in yeast, we therefore constructed a transgene plasmid containing the *CUP1* gene regulated for specific expression in the salivary glands by a promoter of gene coding pig parotid secretory protein. Transgenic CUP1 was highly expressed in the parotid and submandibular salivary glands and secreted in saliva as a 9-kDa copper-chelating protein. Expression of salivary copper-chelating proteins reduced fecal copper contents by 21.61% and increased body-weight by 12.97%, suggesting that chelating proteins improve the utilization and absorbed efficacy of copper. No negative effects on the health of the transgenic mice were found by blood biochemistry and histology analysis. These results demonstrate that the introduction of the salivary *CUP1* transgene into animals offers a possible approach to increase the utilization efficiency of copper and decrease the fecal copper contents.

## Introduction

Copper is an essential trace element and required for survival by a wide range of species, from yeast to mammals [1]. It functions as a cofactor and is required for structural and catalytic properties of a variety of enzymes because of its capacity to act as an intermediary in the transfer of electrons that makes it central to the catalytic activity of the enzymes [2], [3], which are involved in a number of vital biological processes, such as cellular respiration and iron transport, required for growth and development [4]. Thus, Cu supplements were used to treat anemia in animals in the 1920s, and later in chicks [5], pigs [6], infants [7], and adult humans with good success [8].

Feeds provide most of the copper as an essential micronutrient consumed by animals, and drinking water contributes about 6–13% of average daily intake of copper [9], [10], [11], [12]. Most of ingested copper is absorbed in the small intestine, and very small amounts in the stomach [10]. The absorption of copper in the body depends on a variety of factors including its chemical form [10]. Chelated copper has been proven to improve the utilization of copper, which is absorbed more efficiently through an amino acid transport system [13] by increasing intestinal absorption and renal tubular reabsorption of copper, and the chelated form displays increased retention in the body compared with its inorganic form [14], [15], [16], as has been demonstrated in many compounds, such as copper-lysine [17], organic copper chelates [18], copper carbonate [19] and copper-metallothionein (copper-MT) complex [15].

If inorganic coppers are transformed to copper-MT through binding by the MT produced endogenously by animals, such organic copper could be utilized effectively, and then lower doses of inorganic copper could be added in feed, which would, in turn, lead to reduced fecal copper contents. To investigate the feasibility of this hypothesis, we developed transgenic mice that secrete MT proteins in their saliva. The transgenes used in this study contain the *S*. *cerevisiae CUP1* gene. It encodes a Cys-rich protein with a low molecular weight that can bind copper as specifically as copper chelatin in yeast. The protein is characterized principally by its high copper-binding capacity and unusual amino acid composition, and it contains 20% cysteine residues [20], [21]. Mammalian MT is a metal-binding protein that is present in most tissues. The protein was first found to bind cadmium and zinc [22], but it also binds copper and is a major copper-binding protein in the liver, in addition to binding other metals [16]. Yeast CUP1 has highly divergent primary sequences compared to mammalian MT by reconstructing the phylogenetic tree [23]. However, these proteins all possess identical functional sequence motifs, Cys-X-Cys or Cys-X-X-Cys, and binding of copper to these motifs occurs through the Cys residues [21], [24]. In this study, we took advantage of the yeast *CUP1* gene to establish a transgenic mouse model to determine whether endogenous expression of CUP1 can increase copper utilization by mice.

## Materials and Methods

### Ethics Statement

The FVB and ICR mice varieties were used in this research. All animal procedures received approval from the Institutional Animal Care and Use Committee (IACUC) of Shanghai city, China. The mice were housed in the Animal Care Facility at Shanghai Jiao Tong University (IACUC permit numbers: SYXK (Shanghai) 2013-0052).

### Construction of the recombinant plasmids expressing the *CUP1* gene

A fragment, which contained the complete open reading frame (ORF) of the *CUP1* gene, was synthesized based on the published sequence in GenBank (NM_001179185). The vector pPSP (pig parotid secretory protein) was a gift from Ning Li (College of Biological Sciences, China Agricultural University, Beijing, China). The recombinant plasmid pPSP-CUP1 was constructed by insertion of the fragment containing the ORF of the *CUP1* gene, which was digested with *Asc* I (TaKaRa, Japan), into the same endonuclease-digested pPSP vector. The recombinant plasmid was confirmed by restriction analysis and DNA sequencing.

### Transgene purification, quantification and pronuclear microinjection

The linear DNA fragment containing the pPSP promoter, signal peptide and *CUP1* gene was obtained by digestion with *Xho* I and *Not* I and subsequently purified by agarose gel electrophoresis as described by Yin et al. [25]. DNA was resuspended in microinjection buffer, which consisted of 0.1 mmol/L ethylenediaminetetraacetic acid and 10 mmol/L Tris Cl (pH 7.4) at a concentration of 20 ng/µL, and stored at −20°C.

The injection of transgene DNA was performed according to Hogan et al. [26].

### Transgenic examination by PCR and southern blot

The presence of the *CUP1* transgene in the transgenic founders and offspring was confirmed by PCR analysis of genomic DNA derived from tail biopsies and DNA sequencing. PCR was performed with specific primers (forward primer 5′TGTGTAAGCGTGGTAGGTGCTCATC 3′, reverse primer 5′GACACCTACTCAGACAATGCGATGC 3′), and the transgene length was 337 bp. The transgenic founders (G0) were confirmed by Southern blot analysis. Genomic DNA was isolated from the mouse tails using a ZR Genomic DNA-Tissue MiniPrep Kit (Zymo Research, USA). Twenty micrograms of DNA was restriction digested with *Bgl* II and *Ssp* I, fractionated in a 0.8% agarose gel electrophoresis, and transferred to a nylon membrane (Millipore, UK). The fragment containing the complete ORF of yeast *CUP1* was amplified by PCR using specific primers (forward primer 5′TGGGGAATCAGTAGGAAGTCTTGGC 3′, reverse primer 5′CCCCAGAATAGAATGACACCTACTC 3′), and the fragment length was 832 bp. The fragment was then purified with Qiagen PCR purification kits before its use as a probe. The membrane was hybridized with a DIG-labeled CUP1 DNA probe (20 ng). Pre-hybridization and hybridization were performed according to the procedures described by Van Rijs et al. [27]. Pre-hybridization was performed for 1 h at 45°C, hybridization for 6 h at 45°C, and then the membrane was washed twice in 2×standard saline citrate (SSC), 0.1% SDS at 65°C for 20 min, and 0.5×SSC with 0.1% SDS once. Hybridization signals were examined using a Roche DIG DNA Labeling and Detection Kit according to the manufacturer's instructions.

### Western blot analysis

Polyclonal antibodies (Santa Cruz Biotechnology, USA) were raised against amino acids 1–61 taken from the CUP1 and represented the full-length CUP1 sequence of *S. cerevisiae*. The β-actin antibodies were purchased from Sigma-Aldrich (St Louis, MO). Western blot analysis was performed according to the methods of Spencer et al. [28].

Approximately 200 µL of saliva per mouse was collected from 6-wk-old mice as described by Hu et al. [29] and stored at -80°C. The proteins were extracted from the tissues and saliva by homogenization in lysis buffer, separated by SDS-PAGE electrophoresis, and transferred to nitrocellulose. Immunoblotting was performed with antibodies against CUP1 (1∶400) and β-actin (1∶3000), which served as loading controls. As the secondary antibody, a goat anti-rabbit horseradish peroxidase-conjugated antibody was diluted to 1∶5000.

### Detection of the copper content in mouse manure and changes of body weight

Prepared feed was purchased from Shanghai SLAC Laboratory Animal Co., Ltd., China. The feed contained 10 mg/kg content of copper, which is in concordance with the national nutrition standard GB 14924.3–2010 [30].

The G1 offspring were weaned at 4 wk of age, and the transgenic mice were confirmed as described above. Then, the transgenic and control mice were individually caged under controlled temperature (22±2°C), humidity (40–60%) and lighting (12 h light; 12 h darkness) and fed the prepared feed and water ad libitum. Body weight was recorded once daily for 2 wk. Fecal samples were collected once daily for 2 wk and then placed into 2 mL sterile tubes and dried immediately. The samples were dried (130°C) for 48 h and ashed at 600°C for 4 h in a muffle furnace. Next, they were cooled, weighed, and digested in nitric acid (Merck, Germany) at 95°C for at least 2 h. After filtration, the contents of copper in mouse manure were measured by ICP-MS (7500 Series ICP-MS system; USA). Each digested sample volume was standardized to 5 mL.

### Blood biochemistry and histology analysis

Blood samples of approximately 1 mL per mouse were obtained from the retro-orbital venous plexus of the transgenic and control mice using heparinized capillary tubes. Five mice at 6 wk of age and ten mice at 1 yr of age in each group were used for the blood biochemistry analysis.

The blood samples were centrifuged at 3000 rpm for 10 min for the sera. The sera were stored at −80°C prior to blood biochemistry analysis. Nineteen blood biochemical parameters, including Ca (Calcium ion), Fe (ferrum ion), GLU (glucose), CRE (creatinine), CHO (cholesterol), BUN (blood urea nitrogen), AMY (amylase), ALT (alanine aminotransferase), AST (aspartate aminotrasferase), and ALP (alkaline phosphatase), were detected using an auto-analyzer (Hitachi 7180, Hitachi, Japan).

After blood drawing, the mice were sacrificed for histopathology analysis. Tissues (heart, liver, spleen, stomach, kidney, intestine, brain, parotid gland and submandibular gland) were collected and fixed in PBS buffered 10% formalin. The specimens, after paraffin embedding, were sectioned horizontally at 5 µm thickness, stained with hematoxylin and eosinaccording to standard protocol, and observed using a microscope (Nikon, Japan) at an excitation wavelength of 559 nm.

### Statistical analysis

The phenotypic data (the fecal ash copper contents and the body-weight increases of transgenic and control mice) were analyzed separately based on a general linear model (SAS 9.3): y*_ijk_* = *µ*+s*_i_*+d*_j_*+g*_k_*+*e_ijk_* Where

y*_ijk:_* the phenotypic value

*µ*: an overall mean

s*_i_*: a fixed paternal effect

d*_j_*: a fixed maternal effect

g*_k_*: a fixed CUP1 gene effect

*e_ijk_*: a residual error effect with a normal distribution N (0, σ^2^)

## Results

### Generation of transgenic mice

The 12.5-kb linear transgene pPSP-CUP1 (construction shown in Fig. S1) was generated by digestion with *Xho* I and *Not* I and introduced into fertilized mouse oocytes through pronuclear injection.

**Figure 1:**
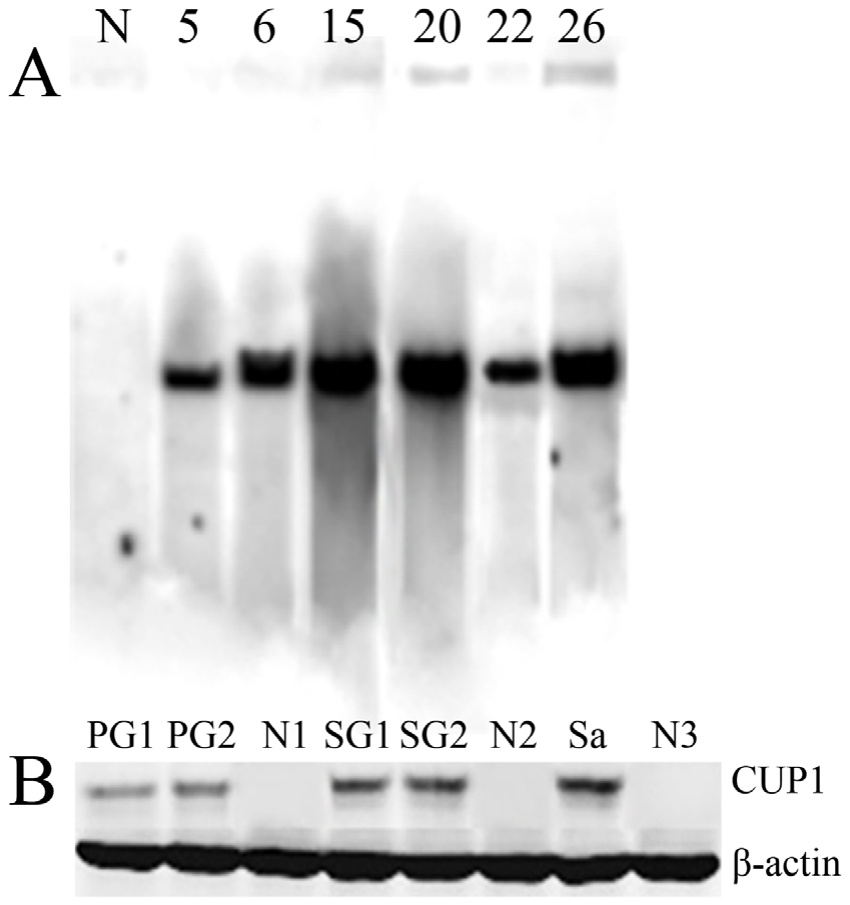
Identification of yeast *CUP1* transgene by southern blot and western blot analysis. (A) Southern blot analysis of the transgenes. Purified genomic DNA from each transgenic founder was digested with *Bgl* II and *Ssp* I, and analyzed by southern blotting using probes specific for *CUP1*. Transgenic founders numbered 5, 6, 15, 20, 22, and 26; N: genomic DNA of control mice as a negative control. (B) Western blot was performed to confirm expression of CUP1 in the parotid and submandibular glands and in the saliva of transgenic lines. The expected protein size is 9 kDa. The lower band is β-actin (42 kDa, used as an internal control). The results demonstrated high expression of CUP1 in the parotid and submandibular glands after normalization against β-actin, as well as that in the saliva of transgenic mice. PG1 and PG2: the parotid glands of transgenic mice; SG1 and SG2: the submandibular glands of transgenic mice; Sa: the saliva of transgenic mice; N1, N2, and N3: the parotid gland, the submandibular gland and the saliva of control mice as negative controls, respectively.

Four male (No. 5, 6: FVB mice; No. 20, 22: ICR mice) and two female (No. 15: FVB mouse; No. 26: ICR mouse) transgenic founder (G0) mice obtained from 29 mice were confirmed by PCR screening and DNA sequencing. Southern blotting was further used to identify the transgene integrated into the genome of the transgenic mice. The transgenes shares the same 832-bp sequences containing the complete ORF of yeast *CUP1* as the probe. The results indicated that the transgene was integrated into the genome (Fig. 1A). Six transgenic founders were mated twice with wild-type mice, of which 4 males transmitted the transgene to their offspring, and 2 females did not pass the transgene to their progeny. A total of 46 G1 transgenic mice were confirmed by PCR amplification from genomic DNA and sequencing among 77 offspring (Table S1).

### Expression of yeast *CUP1* transgene in the salivary glands

The *CUP1* transgene mRNA expression in the salivary glands of transgenic founder was analyzed by reverse transcription PCR. The results revealed that *CUP1* was expressed in the parotid and submandibular glands and was barely expressed in the heart, liver, spleen, stomach, kidney, intestine, and brain tissue of the transgenic founders. However, the CUP1 gene was not expressed in all tissues of the control mice (Fig. S2).

The CUP1 protein was detected in the parotid and submandibular glands using anti-CUP1 antibodies to probe western blot analysis in transgenic founders. The level of β-actin in each sample was determined as the control for protein loading. The results indicated the presence of CUP1 in both detected tissues and indicated relatively high expression in the submandibular glands after normalization against β-actin. In contrast, relatively low expression was observed in the parotid glands (Fig. 1B). CUP1 protein was also detected in the salivary fluid of the transgenic mice by western blot analysis (Fig. 1B), and no CUP1 protein was detected in the saliva of the control mice. The molecular mass of the protein containing CUP1 was 9 kDa as identified in the salivary glands and the secreted saliva.

### The contents of copper in mouse manure ash and changes of body weight

**Figure 2:**
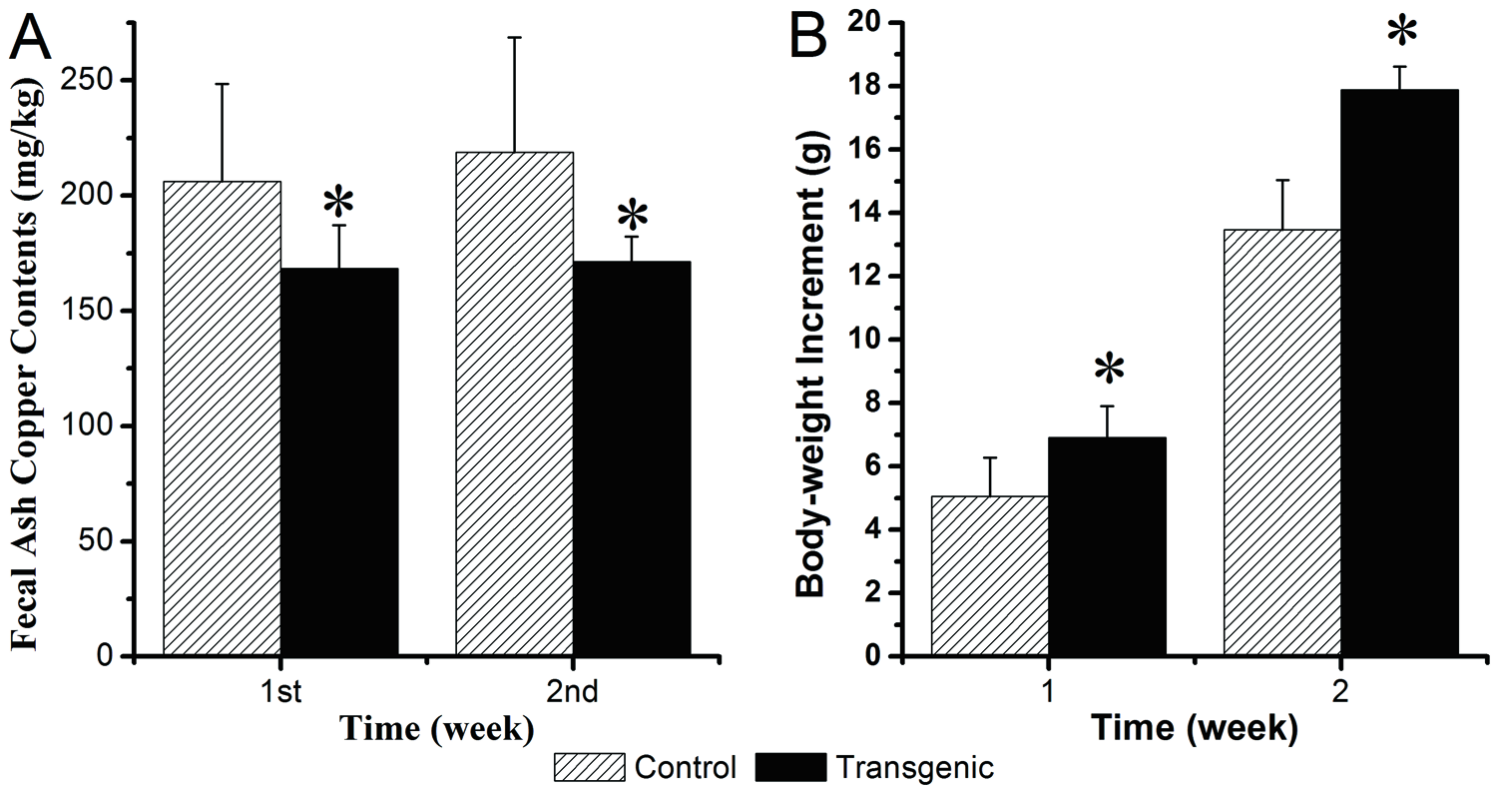
Fecal ash copper contents and changes of body weight of the transgenic and control mice. At 4 wk of age, all G1 offspring were weaned and fed with the prepared feed (copper content, 10 mg/kg). Subsequently, the fecal samples were collected and the body weights of the mice were recorded once daily for 2 wk. (A) The fecal copper contents of the mice were analyzed. The fecal ash copper contents of transgenic mice (n = 28) were significantly lower than the control mice (n = 16), presenting a reduction of 18.41% (*P = 0.0022<0.01) and 21.61% (*P = 0.0003<0.01) for the first week and the second week, respectively. (B) The changes of body weight of the mice were analyzed. After 1 and 2 wk, the body-weight of transgenic mice (n = 28) increased significantly (*P = 0.025<0.05; *P = 0.019<0.05, respectively) compared with those of the control mice (n = 16) at 7.2% and 12.97%, respectively. 1st: the first; 2nd: the second.

Forty-four G1 offspring were selected from the total G1 mice considering similar weights and used for further experiments, of which 28 were transgenic mice, and 16 were control mice. To determine the effect of the expressed CUP1 on the transgenic mice, the usage efficiency of copper in the prepared feed was investigated by detecting the contents of copper in mouse manure from the transgenic and control mice at a dietary level of 10 mg/kg copper. For the first week, the transgenic mice exhibited manure ash copper contents of 168.285±18.849 mg/kg, a reduction of 18.41% (*P* = 0.0022<0.01) compared with the control mice (206.263±42.307 mg/kg) raised under the same conditions. At the second week, the manure ash copper contents of transgenic mice (171.449±10.767 mg/kg) were significantly lower (*P* = 0.0003<0.01) compared with that (218.713±49.831 mg/kg) of the control mice. This represents a reduction of 21.61% in the ash copper contents under the same conditions (Fig. 2A). The effect of the expressed CUP1 on body weight of the transgenic mice was also analyzed. At day 0, the body weight of the control group was 20.531±1.099 g and that of the transgenic group was 20.835±1.214 g. After 1 wk, the body-weight increases of the transgenic mice (6.906±0.998 g) were significantly greater (*P* = 0.025<0.05) compared with those (5.063±1.214 g) of the control mice (Fig. 2B). On average, the transgenic mice were 7.2% heavier than the control mice raised under the same conditions. After 2 wk, the body-weight increases of the transgenic mice (17.884±0.728 g) were significantly greater (*P* = 0.019<0.05) compared with those (13.475±1.556 g) of the control mice (Fig. 2B). On average, the transgenic mice were 12.97% heavier than the control mice raised under the same conditions.

### Analysis of blood biochemistry and histology

The blood biochemistry results revealed that the levels of GLU, BUN, AMY, ALT, AST, and ALP in serum were slightly elevated in the transgenic mice at 6 wk of age compared with the control mice. In contrast, the blood concentration of CRE was slightly decreased, and all differences were not significant (*P*>0.05; Table 1). The similar results were observed in the mice at 1 yr of age, but the level of ALP was slightly decreased in the transgenic mice (Table S2). Differences between the transgenic and control mice were hardly observed for other serum biochemical parameters.

**Table 1:** Blood biochemistry results in the transgenic and control mice at 6 wk of age.

	ALB (g/L)	GLOB (g/L)	A/G	TP (g/L)	GLU (mmol/L)	CHO (mmol/L)	TG (mmol/L)
Transgenic	18.167±1.002	24.333±0.577	0.757±0.038	42.2±1.323	5.165±0.332	2.133±0.153	1.3±0.386
Control	18.400±1.365	24.5±2.082	0.7±0.026	42.967±3.362	4.335±0.458	1.717±0.097	1.347±0.375

	Ca (mmol/L)	Fe (mmol/L)	HDL (mmol/L)	LDL (mmol/L)	UA (µmol/L)	BUN (mmol/L)	CRE (µmol/L)
Transgenic	1.58±0.115	36.833±1.501	1.513±0.101	0.463±0.148	187.5±25.03	6.6±0.917	3.145±0.518
Control	1.433±0.076	33.767±3.465	1.533±0.163	0.427±0.154	176.867±15.689	5.567±0.586	6.76±0.679

	LDH (U/L)	AMY (U/L)	ALT (U/L)	AST (U/L)	ALP (U/L)
Transgenic	1165.867±161.645	1801.43±195.493	35.433±3.047	130.633±15.314	64.5±8.839
Control	1173.167±97.779	1627.097±94.849	29.6±0.566	105.467±10.919	51.7±2.83

*The differences between the transgenic (n = 5) and control mice (n = 5) were not significant (P>0.05) for all examined serum biochemical parameters.*

ALB: albumin; GLOB: globulin; A/G: ALB/GLOB; TP: total protein; GLU: glucose; CHO: cholesterol; TG: triglyceride; Ca: calcium ion; Fe: ferrum ion; HDL: high density lipoprotein; LDL: low-lipid lipoprotein; UA: uric acid; BUN: blood urea nitrogen; CRE: creatinine; LDH: lactate dehydrogenase; AMY: amylase; ALT: alanine aminotransferase; AST: aspartate aminotrasferase; ALP: alkaline phosphatase.

**Figure 3:**
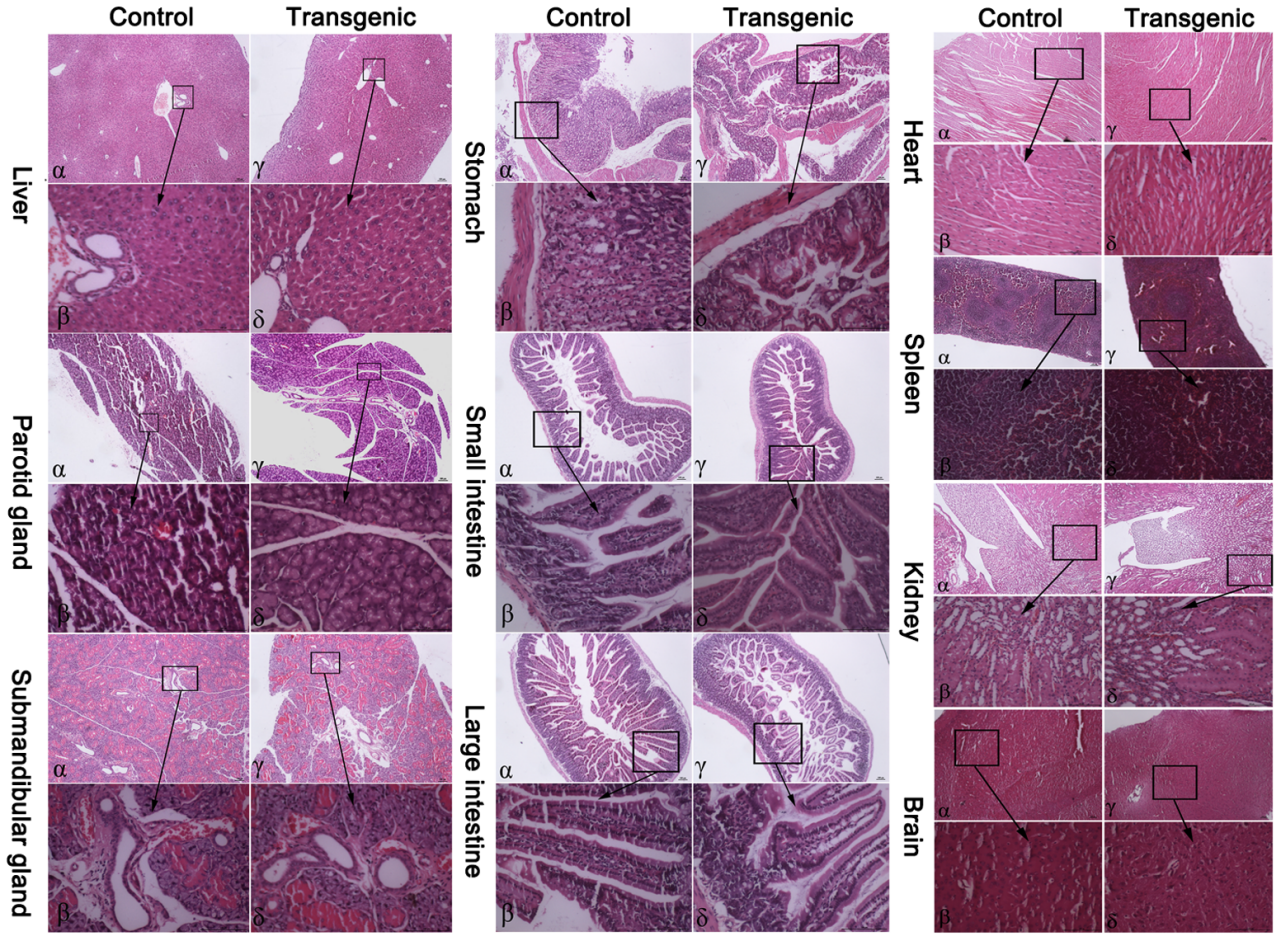
Histological analysis of the tissues of the transgenic and control mice. The heart, liver, spleen, stomach, kidney, small intestine, large intestine, brain, parotid gland, and submandibular gland tissue samples from the transgenic mice (transgenic; n = 5) and control mice (n = 5) at 6 wk of age were analyzed by histology observation. In the above pictures, α and γ are whole tissues, and β and δ are amplified regions of the tissues. The length of the scale bar is 100 µm in all micrographs. The profiles of the tissues of the transgenic and control mice were determined. No obvious changes were observed in the tissues of the transgenic mice compared with those of the control mice.

At the ages of 6 wk and 1 yr, the transgenic mice were in good health and did not exhibit any gross pathological abnormalities or illness. Histological analysis was performed in the tissues (heart, liver, spleen, stomach, kidney, intestine, brain, parotid gland, and submandibular gland) of the mice, and no obvious changes were observed in the tissues of the transgenic mice compared with those of the control mice, and the results were shown in Fig. 3 and Fig. S3, respectively.

## Discussion

Metallothionein is a highly conserved family of closely related proteins.

Yeast CUP1 is a member of the MT family and accounts for copper-binding in *S. cerevisiae* [31]. Mammals have the *MT* gene, and it possesses multiple isoforms [32]. In this study, we took advantage of yeast *CUP1* for transgene as it shares functional sequence identity to mammalian *MT*. Thus, yeast CUP1 binds to copper through Cys residues by the formation of metal-thiolate linkages, as well as the mammalian MT proteins [33], suggesting that this gene may be effective in copper-binding in mammals. This gene has been used for transgene in some organisms. Yeast *CUP1* was introduced to tobacco plants, and its expression contributed to copper content because of its role in copper-binding [32]. In *Drosophila*, this gene was selected as the transgene instead of the endogenous *Mtn* gene, which has a similar structure and function with yeast *CUP1*, to determine its role in binding copper [34].

Many researchers have used the promoter of the mouse salivary gland-specific *PSP* gene to express the exogenous genes such as *phytase* gene in the saliva of transgenic mice, which has been confirmed to be feasible [25], [35], [36]. A similar phenomenon was examined in our results, and constitutive expression in the PSP/CUP1 mice was notably specific for the parotid and submandibular glands. A three-fold higher expression was detected in submandibular glands compared with the parotid glands, and a similar phenomenon was detected by Mikkelsen et al. (1992) [37], and the opposite observation was reported by Golavan et al. (2001) [36].

Copper as a feed additive is effective in growth enhancement and disease prevention in weanling pigs, and it is widely used in pork production around the world, especially in China [38], [39]. However, copper in pig diets heavily exceeds the minimum requirements for normal performance (5–25 mg/kg copper for different classes of pigs) [40], and most of the indigested copper, acting as promotants, by pigs is excreted in the manure (>90%) [41]. The concentrations of copper in pig manure are 5–12 times those in pig feeds with additives [42], which are higher than for other agricultural animals, such as cattle and sheep [43]. The application of pig manure directly onto agricultural land as fertilizer is common practice in China [39]. Because of copper's low mobility and non-degradation, copper can accumulate in soils [44], which leads to environmental consequences. When manure is repeatedly applied as fertilizer, copper can cause surface pollution with severe biological consequences, e.g., causing toxicity to plants, elevating bacterial resistance to toxic metals and increasing human exposure to copper via the food chain [39], [40], [45]. Still, the present inputs of copper are too high and reducing the contents of copper in the diet should reduce concentrations in the pig manure [46]. In the present study, we validated a method to increase copper utilization efficiency and to decrease the fecal copper content by providing the animals with an exogenous gene for generation of copper chelatin in the saliva. We determined that this approach can reduce mouse fecal copper content by 21.61%. Therefore, this might provide an important clue for preventing the pollution caused by the fecal copper in the pig production.

In addition, we reduced the dietary supply of copper with a 10 mg/kg concentration and demonstrated its feasibility for decreasing fecal copper content. Similar results were observed by Jondreville et al. (2003) [47]. The *CUP1* transgene mice, at a dietary level of 10 mg/kg copper, displayed a body weight-increase response, with the transgene mice 12.97% heavier compared with the control mice after 2 wk. These results are similar to those obtained when animals were fed 250 mg/kg of dietary copper [48]. The decreased fecal copper contents and increased body-weight increases caused by the transgene yeast CUP1 suggest that the *CUP1* transgene most likely enhanced the usage of copper in the diet. Copper is able to stimulate the secretion of several neuropeptides and growth hormones [49], in addition to being a component of the growth factor Iamin [48]. Therefore, copper could influence the growth regulatory system in many ways and might be the main reason for the growth stimulation.

No significant difference was found in a range of markers and the histology of tissues of the transgenic mice compared with the controls. These results suggest that the *CUP1* transgene did not affect the blood composition and histology of the mice. The transgenic mice were confirmed to be in good health and did not exhibit any gross pathological abnormalities or illness.

In summary, we have demonstrated that the repertoire of copper-chelating proteins produced by a model animal can be modified by introduction of *CUP1* transgene into its genome. The salivary copper-chelating proteins in these mice lead to a significant reduction of fecal copper levels and a significant increase of body weight, suggesting the enhancement of the utilization efficiency of the dietary copper by transgenic mice. Our findings provide the essential data toward elucidating the physiological functions of *MT* gene on copper metabolism.

## Supporting Information

Figure S1 **Construction of the recombinant plasmids expressing** ***CUP1*** **gene and confirmation by PCR and restriction.** (A) The recombinant plasmid pPSP-CUP1 was constructed by insertion of the fragment containing the ORF of the *CUP1* gene into the same endonuclease-digested pPSP vector. (B) The recombinant plasmid was confirmed by restriction analysis, DNA sequencing, and by PCR.
(TIF)

Figure S2 **RT-PCR analysis of yeast** ***CUP1*** **transgene expression.** (A, B) The *CUP1* transgene mRNA expression was analyzed by RT-PCR in the salivary glands of the transgenic founders and the control mice, respectively.
(TIF)

Figure S3 **Histological analysis of the tissues of the transgenic and control mice at 1 yr of age.** The heart, liver, spleen, stomach, kidney, small intestine, large intestine, brain, parotid gland, and submandibular gland tissue samples from the transgenic mice (transgenic; n = 10) and control mice (n = 10) at 1 yr of age were analyzed by histology observation. In the above pictures, α and γ are whole tissues, and β and δ are amplified regions of the tissues. The length of the scale bar is 100 µm in all micrographs. The profiles of the tissues of the transgenic and control mice were determined. No obvious changes were observed in the tissues of the transgenic mice compared with those of the control mice.
(TIF)

Table S1 **Generation of G1 transgenic mice.**
(DOCX)

Table S2 **Blood biochemistry results in the transgenic and control mice at 1 yr of age.**
(DOCX)
