# Supplementary figures and images for: Transgenic Mice Expressing Yeast CUP1 Exhibit Increased Copper Utilization from Feeds
Source: PLoS One. 2014 Sep 29;9(9):e107810. doi: 10.1371/journal.pone.0107810 (PMC4180272; doi:10.1371/journal.pone.0107810)

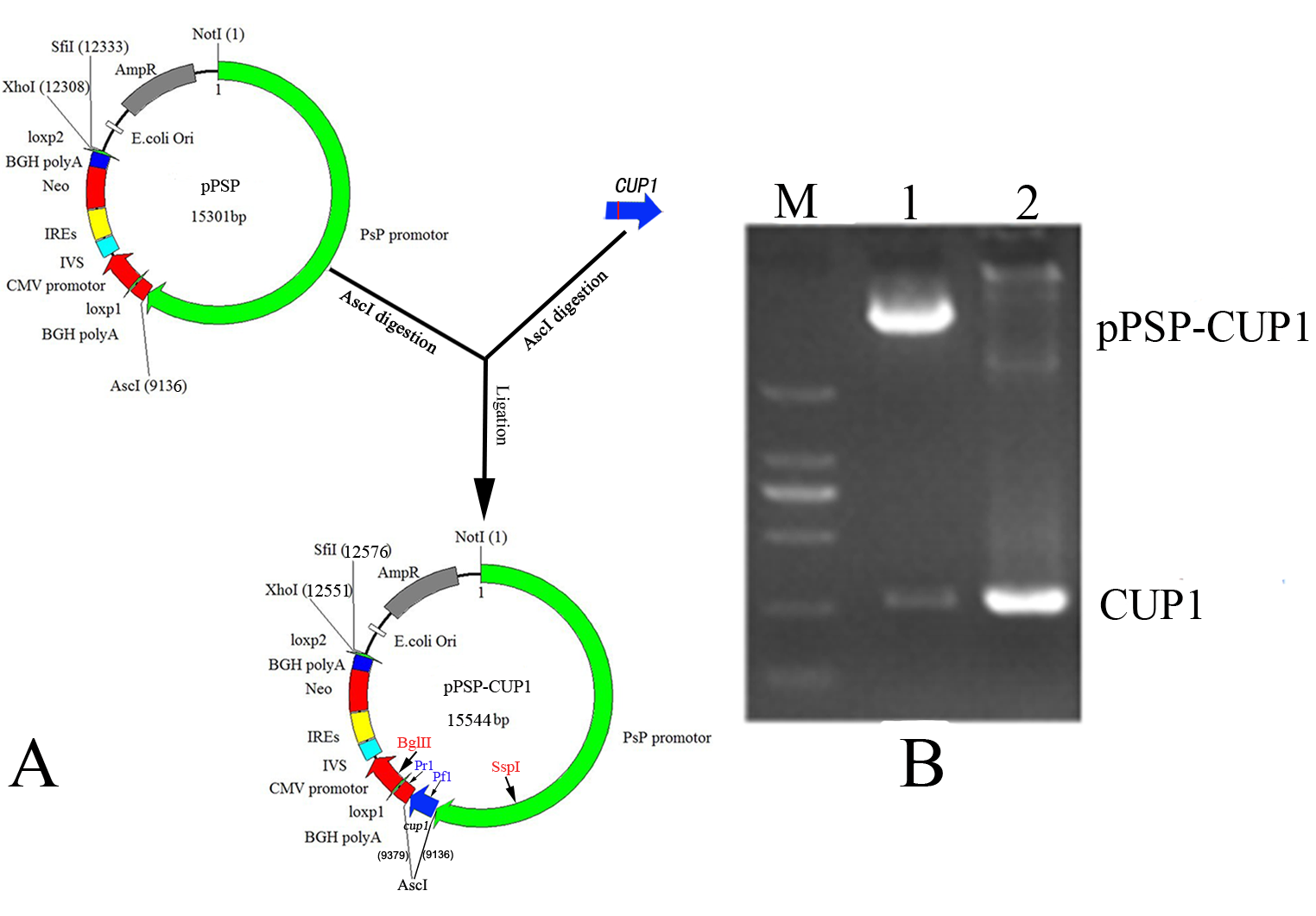

Supplement: Figure S1 — Construction of the recombinant plasmids expressing CUP1 gene and confirmation by PCR and restriction. (A) The recombinant plasmid pPSP-CUP1 was constructed by insertion of the fragment containing the ORF of the CUP1 gene into the same endonuclease-digested pPSP vector. (B) The recombinant plasmid was confirmed by restriction analysis, DNA sequencing, and by PCR. (TIF) [file pone.0107810.s001.tif]

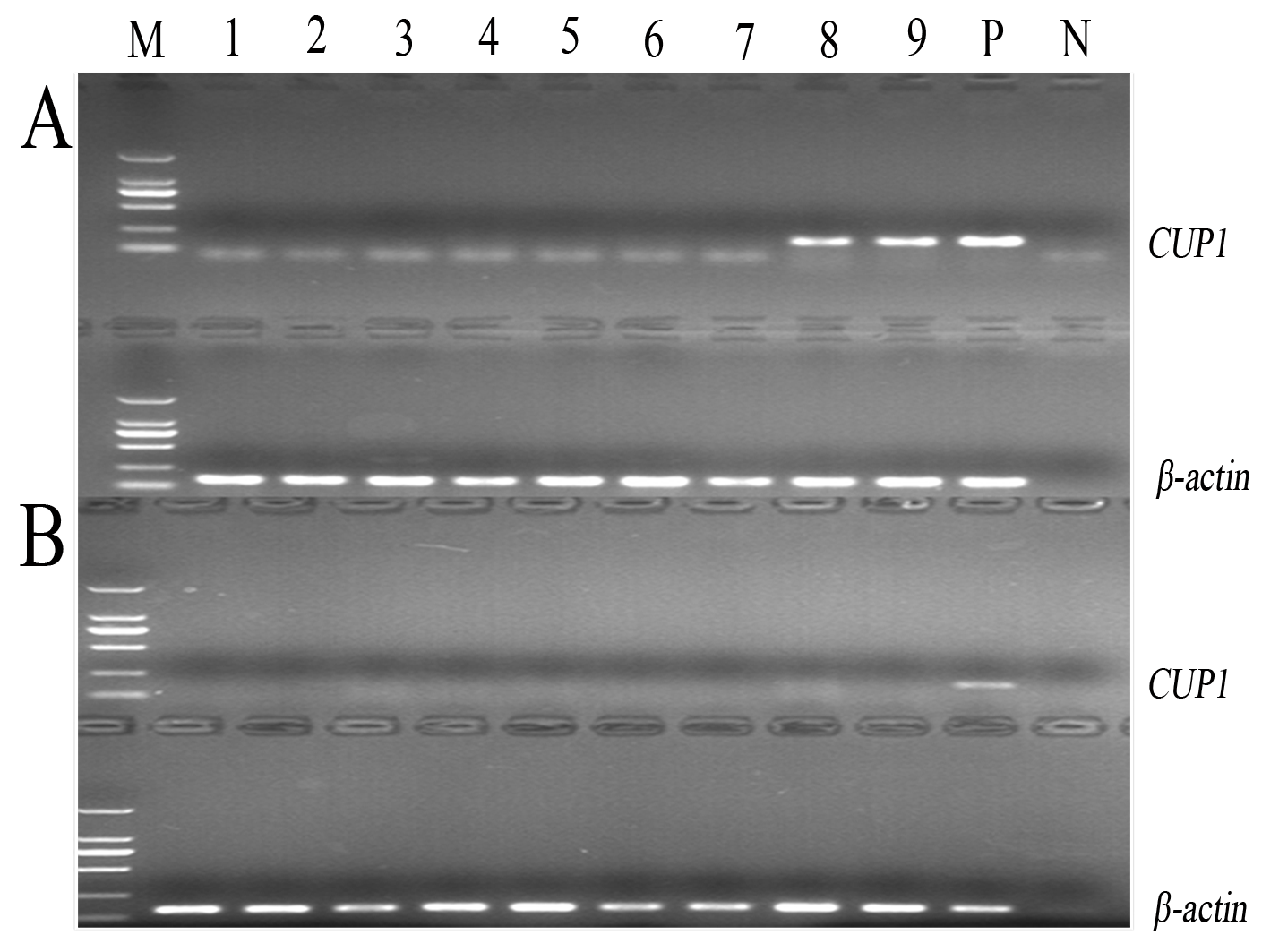

Supplement: Figure S2 — RT-PCR analysis of yeast CUP1 transgene expression. (A, B) The CUP1 transgene mRNA expression was analyzed by RT-PCR in the salivary glands of the transgenic founders and the control mice, respectively. (TIF) [file pone.0107810.s002.tif]

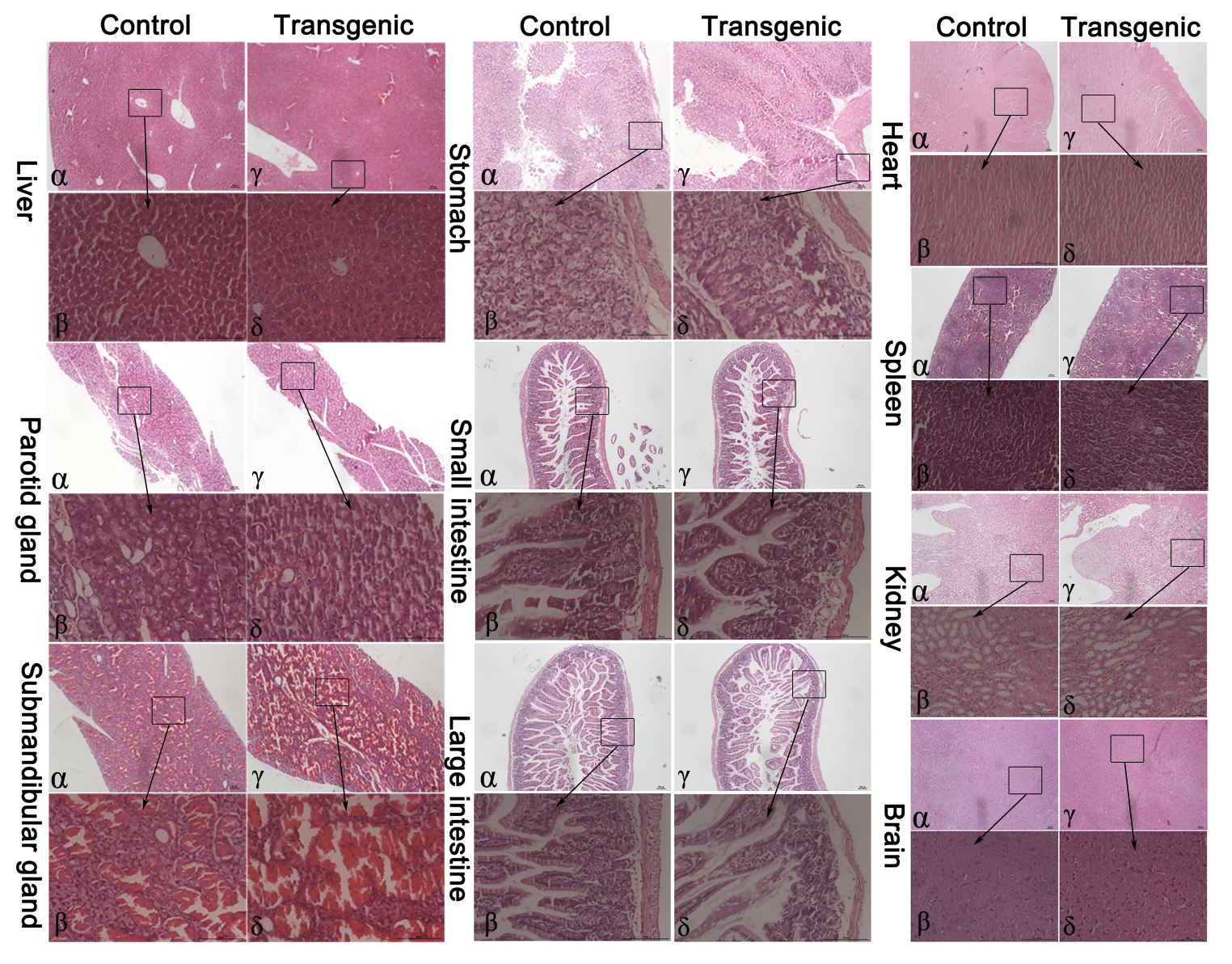

Supplement: Figure S3 — Histological analysis of the tissues of the transgenic and control mice at 1 yr of age. The heart, liver, spleen, stomach, kidney, small intestine, large intestine, brain, parotid gland, and submandibular gland tissue samples from the transgenic mice (transgenic; n = 10) and control mice (n = 10) at 1 yr of age were analyzed by histology observation. In the above pictures, α and γ are whole tissues, and β and δ are amplified regions of the tissues. The length of the scale bar is 100 µm in all micrographs. The profiles of the tissues of the transgenic and control mice were determined. No obvious changes were observed in the tissues of the transgenic mice compared with those of the control mice. (TIF) [file pone.0107810.s003.tif]
