# Supplementary material for: Transgenic Mice Expressing Yeast CUP1 Exhibit Increased Copper Utilization from Feeds
Source: PLoS One. 2014 Sep 29;9(9):e107810. doi: 10.1371/journal.pone.0107810 (PMC4180272; doi:10.1371/journal.pone.0107810)
Supplement: Table S1 — Generation of G1 transgenic mice. (DOCX) [file pone.0107810.s004.docx]

**Table S1 Generation of G1 transgenic mice**

| Transgenic vectors | No. of G0 | Breeding  batch | Quantity of G1 mice born | Quantity of transgenic mice | Ratio of transgene |
| --- | --- | --- | --- | --- | --- |
| pPSP-CUP1 | 5 (♂) | 1 | 10(4♂6♀) | 6(3♂3♀) | 60% |
|  | 6 (♂) | 1 | 14(7♂7♀) | 6(1♂5♀) | 42.85% |
|  |  | 2 | 8(7♂1♀) | 5(4♂1♀) | 62.5% |
|  | 20 (♂) | 1 | 14(5♂9♀) | 8(3♂5♀) | 57.14% |
|  | 22 (♂) | 1 | 20(5♂4♀) | 13(8♂5♀) | 65% |
|  |  | 2 | 11(8♂3♀) | 8(5♂3♀) | 72.72% |

G1: Generation 1.
