# Supplementary material for: Transgenic Mice Expressing Yeast CUP1 Exhibit Increased Copper Utilization from Feeds
Source: PLoS One. 2014 Sep 29;9(9):e107810. doi: 10.1371/journal.pone.0107810 (PMC4180272; doi:10.1371/journal.pone.0107810)
Supplement: Table S2 — Blood biochemistry results in the transgenic and control mice at 1 yr of age. (DOCX) [file pone.0107810.s005.docx]

**Table S2 Blood biochemistry results in the transgenic and control mice at 1 yr of age**

|  | ALB (g/L) | GLOB (g/L) | A/G | TP (g/L) | GLU (mmol/L) | CHO (mmol/L) | TG (mmol/L) |
| --- | --- | --- | --- | --- | --- | --- | --- |
| Transgenic | 17.253±1.110 | 25.25±0.871 | 0.704±0.085 | 43.07±2.104 | 5.714±0.752 | 1.776±0.22 | 1.187±0.33 |
| Control | 17.481±1.113 | 24.91±1.269 | 0.712±0.039 | 42.17±2.132 | 5.453±0.875 | 1.904±0.056 | 1.145±0.341 |
|  | Ca (mmol/L) | Fe (mmol/L) | HDL (mmol/L) | LDL (mmol/L) | UA (μmol/L) | BUN (mmol/L) | CRE (μmol/L) |
| Transgenic | 1.517±0.128 | 34.25±1.543 | 1.448±0.198 | 0.584±0.124 | 202.67±28.765 | 6.76±1.173 | 3.846±0.728 |
| Control | 1.504±0.127 | 31.98±4.728 | 1.564±0.158 | 0.533±0.168 | 193.591±13.768 | 6.4±0.937 | 4.919±0.676 |
|  | LDH (U/L) | AMY (U/L) | ALT (U/L) | AST (U/L) | ALP (U/L) |  |  |
| Transgenic | 1213.27±143.29 | 1788.162±204.162 | 34.77±2.61 | 135±15.127 | 49.74±1.68 |  |  |
| Control | 1173.13±76.731 | 1740.592±80.397 | 32.891±3.93 | 126.164±13.971 | 54.927±1.335 |  |  |

*The differences between the transgenic (n=10) and control mice (n=10) were not significant (P>0.05) for all examined serum biochemical parameters.*

ALB: albumin; GLOB: globulin; A/G: ALB/ GLOB; TP: total protein; GLU: glucose; CHO: cholesterol; TG: triglyceride; Ca: calcium ion; Fe: ferrum ion; HDL: high density lipoprotein; LDL: low-lipid lipoprotein; UA: uric acid; BUN: blood urea nitrogen; CRE: creatinine; LDH: lactate dehydrogenase; AMY: amylase; ALT: alanine aminotransferase; AST: aspartate aminotrasferase; ALP: alkaline phosphatase.
